# A hybrid level set model for image segmentation

**DOI:** 10.1371/journal.pone.0251914

**Published:** 2021-06-07

**Authors:** Weiqin Chen, Changjiang Liu, Anup Basu, Bin Pan

**Affiliations:** 1 Artificial Intelligence Key Laboratory of Sichuan Province, Automation and Information Engineering, Sichuan University of Science and Engineering, Zigong, China; 2 College of Automation and Information Engineering, Sichuan University of Science and Engineering, Zigong, China; 3 College of Mathematics and Statistics, Sichuan University of Science and Engineering, Zigong, China; 4 Department of Computing Science, University of Alberta, Edmonton, Canada; Wuhan University of Science and Technology, CHINA

## Abstract

Active contour models driven by local binary fitting energy can segment images with inhomogeneous intensity, while being prone to falling into a local minima. However, the segmentation result largely depends on the location of the initial contour. We propose an active contour model with global and local image information. The local information of the model is obtained by bilateral filters, which can also enhance the edge information while smoothing the image. The local fitting centers are calculated before the contour evolution, which can alleviate the iterative process and achieve fast image segmentation. The global information of the model is obtained by simplifying the C-V model, which can assist contour evolution, thereby increasing accuracy. Experimental results show that our algorithm is insensitive to the initial contour position, and has higher precision and speed.

## 1 Introduction

With the development of electronic computers, as well as the extensive practical needs in military, industry, and medicine, the field of digital image processing has emerged [1–4]. People use computers to process graphics and images, and segment the target Region of Interest (RoI) in digital images. Image segmentation has a direct impact on subsequent operations, such as image recognition, analysis and understanding [5–7].

Among existing segmentation methods, the active contour model (ACM) [8] has unique advantages. This technique minimizes an energy function to drive the initial contour to reach the boundary of the target region, to extract the RoIs. According to the different contour construction modes, it can be divided into parametric active contour model [8–13] and geometric active contour model [14–35].

In 1998, Osher [36] proposed the level set method, which presents the closed contour in an implicit way. This avoids tracking the contour evolution process, transforms the contour evolution into a pure partial differential equation (PDE) solution, and solves the problem of splitting and merging of contours. However, the level set method is numerically unstable after contour evolution for a period of time. Reinitialization results in higher computation time and slower contour evolution rate. Caselles et al. [22] and Malladi et al. [23] combined level sets with the active contour model on the basis of the predecessors, proposed the geometric active contour model based on level sets, and created the classical geodesic active contour (GAC) model. Subsequently, many scholars began to combine the active contour model with level sets for image segmentation, for automatic processing of topological changes. In 2001, Chan and Vese (C-V) [24] proposed an active contour model without edge information by simplifying the M.S. model. This model abandons the gradient stopping criterion and adopts two piecewise constants as the fitting centers. It has the advantages of area-based methods and performs well for noisy images and images whose edges are difficult to detect. Nevertheless, the energy functional only contains global information. Thus, the C-V model is not good for grayscale unbalanced images. In order to solve the problem of reinitialization of the level set function, Li et al. [25] first proposed a geometric active contour model without reinitialization, The internal energy term in the model, also called penalty term, controls the level set gradient to stabilize near 1, This keeps the level set function as a symbol distance function in the evolution process, and does not need to be reinitialized. Li et al. proposed the local binary fitting model (LBF) model [26], which has a good segmentation effect for images with uneven grayscale. However, it is very sensitive to initialization. Thus, repeat trials are needed to find an appropriate initial contour. Based on local information, Zhang et al. [27] proposed active contours driven by local image fitting energy (LIF) with higher performance. But it still has initialization sensitivity and is inclined to fall into a local minima.

In recent years, many scholars consider more image information and constraints, proposed many better hybrid models [28–32], which solve the problem for the segmentation of images with intensity inhomogeneity in some extent. By combining the local region-based ACMS (LR-ACMs) and global region-based ACMs (GR-ACMs) with a weight coefficient, the shortcomings of GR-ACMS in weak edge image segmentation and LR-ACMS in poor robustness are avoided. In such a combination, the advantages of the GR-ACMs in high robustness and of the LR-ACMs in detecting the weak boundaries of objects are preserved. Classical methods include local and global strength fitting (LGIF) models [28], the global and local region active contour (GARAC) [30]. Ding et al. [31] constructed an optimization Laplace of Gaussian energy for image segmentation, called LOGF. In [33], the author regularized the level set by using a new diffusion function, and proposed a new edge indicator function to resist the noise of the image. Yu et al. [34] constructed a new local region model using bilateral filtering, which has a significant effect on images with no obvious boundary. Liu et al. [35] designed a global pressure force based on symbolic energy (GLSE) to improve its robustness against the initial curve.

In this paper, we propose an active contour model combining global and local fitting. The energy function of the model consists of a global fitting term, a local fitting term and a regularization term. First, we simplify the piecewise smoothing model and define the global fitting function. Second, a local grayscale weighted fitting function is proposed as the fitting center. The fitting function is defined as the local grayscale weighted average image intensity before evolution. Finally, the global fitting term is combined with the local fitting term, and a regularization term is incorporated to ensure smoothing and no re-initialization. The steepest descent method is used to solve the model. The global fitting and local fitting working together to drive the contour to the target boundary. The regularization term guarantees numerical stability. Experimental results show that the proposed model has good segmentation performance for simple images, noisy images and inhomogeneous images.

The rest of this paper is organized as follows: in the next section, we briefly review some classical models, including C-V model [24], LBF model [26], LIF model [27], and the active contour model driven by local and global intensity fitting energy (LGIF) [28]. In Section 2, we describe the structure of the proposed model in detail. Experimental results and analysis are given in Section 3. Finally, concluding remarks are given in Section 4.

## 2 Background

### 2.1 C-V model

Chan and Vese [24] proposed the famous borderless active contour model. Assuming that the gray level of an individual region of an image is homogeneous, for a given image *I*_0_(*x*, *y*), (*x*, *y*) ∈ Ω, is divided by a closed contour *C* into internal and external area, namely Ω_1_ and Ω_2_, respectively. Henceforth, *c*_1_, *c*_2_ are the average grey values of Ω_1_, Ω_2_, then the energy functional structure of C-V model in the form of a level set function *ϕ* is defined as:

$$\begin{array}{c} \begin{array}{cc} E(c_{1},c_{2},\phi ) & =\mu \int_{\Omega }\delta_{\varepsilon }(\phi )|\nabla \phi |dx\\ & +\lambda_{1}\int_{\Omega }{\left|I_{0}-c_{1}\right|}^{2}H_{\varepsilon }(\phi )dx\\ & +\lambda_{2}\int_{\Omega }{\left|I_{0}-c_{2}\right|}^{2}(1-H_{\varepsilon }(\phi ))dx \end{array} \end{array}$$ (1)

where *μ* ≥ 0, λ_1_ > 0, λ_2_ > 0, the *ϕ* is defined as the distance function below:

$$\begin{array}{c} \phi =\{\begin{array}{cc} +d((x,y),C), & (x,y)\in \Omega_{1}\\ 0, & (x,y)\in \Omega \\ -d((x,y),C), & (x,y)\in \Omega_{2} \end{array}\; \end{array}$$ (2)

*H*_*ε*_(*ϕ*) is the heaviside function of *ϕ* in the numeric implementation, and the Dirac function *δ*_*ε*_ is the first derivative of *H*_*ε*_(*ϕ*):

$$\begin{array}{c} \begin{array}{c} \begin{array}{c} H_{\varepsilon }(\phi )=\frac{1}{2}[1+\frac{2}{\pi }\text{arctan}(\frac{\phi }{\varepsilon })]\\ \delta_{\varepsilon }(\phi )=\frac{1}{\pi }.\frac{\varepsilon }{\varepsilon^{2}+\phi^{2}} \end{array} \end{array} \end{array}$$ (3)

Taking the partial derivative of the energy *E* with respect to *c*_1_ and *c*_2_, and setting them to 0, we get the average gray values of *c*_1_, and *c*_2_ as:

$$\begin{array}{c} \begin{array}{cc} c_{1}(\phi ) & =\frac{\int_{\Omega }I_{0}H_{\varepsilon }(\phi )dx}{\int_{\Omega }H_{\varepsilon }(\phi )dx}\\ c_{2}(\phi ) & =\frac{\int_{\Omega }I_{0}(1-H_{\varepsilon }(\phi ))dx}{\int_{\Omega }(1-H_{\varepsilon }(\phi ))dx} \end{array} \end{array}$$ (4)

According to the variational principle, the partial differential equation for *ϕ* is:

$$\begin{array}{c} \begin{array}{c} \frac{\partial \phi }{\partial t}=\delta_{\varepsilon }(\phi )[u\nabla (\frac{\nabla \phi }{\left|\nabla \phi \right|})-\lambda_{1}{\left(I_{0}-c_{1}\right)}^{2}+\lambda_{2}{\left(I_{0}-c_{2}\right)}^{2}] \end{array} \end{array}$$ (5)

The C-V model performs well in images with simple geometric structure and the grayscale equalized image. Also, it is able to segment the image without a gradient defined boundary. While it behaves poorly for uneven gray scale and more complex images, such as the case of target crossings and object occlusion. In addition, the level set function needs to be re-initialized after certain contour updates, which requires a large amount of computation time.

### 2.2 LBF model

In view of the poor performance in images with intensity inhomogeneity for C-V model, Li et al. [26] proposed a local strength fitting energy function:

$$\begin{array}{c} \begin{array}{ccc} & E^{LBF}\left(f_{1},f_{2},\phi \right) & \\ & =\lambda_{1}\int [\int K_{\sigma }\left(x-y\right){\left|I_{0}\left(x\right)-f_{1}\left(x\right)\right|}^{2}H_{\varepsilon }\left(\phi \left(y\right)\right)dy]dx\\ & +\lambda_{2}\int [\int K_{\sigma }\left(x-y\right){\left|I_{0}\left(x\right)-f_{2}\left(x\right)\right|}^{2}\left(1-H_{\varepsilon }\left(\phi \left(y\right)\right)\right)dy]dx \end{array} \end{array}$$ (6)

In (6), the kernel function *K*_*σ*_ is defined as:

$$\begin{array}{c} \begin{array}{c} K_{\sigma }=\frac{1}{{\left(2\pi \right)}^{n/2}\sigma^{n}}e^{-|x^{2}|/2\sigma^{2}} \end{array} \end{array}$$ (7)

*f*_1_, *f*_2_ are two numbers of local intensities, which is calculated as:

$$\begin{array}{c} \begin{array}{cc} f_{1} & =\frac{K_{\sigma }(x)*[H_{\varepsilon }(\phi )I_{0}(x)]}{K_{\sigma }(x)*H_{\varepsilon }(\phi )}\\ f_{2} & =\frac{K_{\sigma }(x)*[\left(1-H_{\varepsilon }(\phi ))I_{0}(x\right)]}{K_{\sigma }(x)*(1-H_{\varepsilon }(\phi ))} \end{array} \end{array}$$ (8)

Because of the localization and characterization kernel function *K*_*σ*_, the fitting center *f*_1_ and *f*_2_ are only affected by the points within a certain range, which is essentially different from *c*_1_ and *c*_2_ in the C-V model. The LBF model solves the problem of the C-V model not being able to segment grayscale non-uniform images. However, because of the gaussian kernel, the energy can easily fall into a local optimal. Thus, the segmentation results depend on the settings of the initial contour. Besides, in the actual calculation, the convolution operation (8) is time consuming.

### 2.3 LIF model

Zhang et al. [27] proposed the Local Image Fitting (LIF) model, with the energy function defined as:

$$\begin{array}{c} \begin{array}{c} E^{LIF}(m_{1},m_{2},\phi )=\frac{1}{2}\int_{\Omega }{\left|I_{0}-I^{LFI}\right|}^{2}dx \end{array} \end{array}$$ (9)

where *E*^*LIF*^ is defined as:

$$\begin{array}{c} \begin{array}{c} \left\{ \begin{array}{c} I^{LFI}=m_{1}(x)H_{\varepsilon }(\phi )-m_{2}(x)(1-H_{\varepsilon }(\phi ))\\ m_{1}(x)=mean(I_{0}(x),x\in \{x\in \Omega |\phi <0\}\cap W_{k}(x))\\ m_{2}(x)=mean(I_{0}(x),x\in \{x\in \Omega |\phi >0\}\cap W_{k}(x)) \end{array}\right. \end{array} \end{array}$$ (10)

where *m*_1_(*x*) and *m*_2_(*x*) can be regarded as the average of the image intensity in the window *W*_*k*_(*x*). Therefore, *m*_1_(*x*) and *m*_2_(*x*) are equivalent to *f*_1_ and *f*_2_ in the LBF model. Utilizing local image information, the LIF model is able to segment images with uneven intensity and only employ half of the convolution operations compared to the LBF model. However, it is still sensitive to initialization, like the LBF model.

### 2.4 LGIF model

Based on the predecessors, Wang et al. [28] introduced the global fitting energy of the C-V model into LBF and proposed a hybrid model in which global and local information working together; with the energy function defined as:

$$\begin{array}{c} \begin{array}{c} E^{LGIF}=(1-\omega )E^{LBF}+\omega E^{GIF} \end{array} \end{array}$$ (11)

where *E*^*GIF*^ is the global fitting energy, consistent with the fitting item in the C-V model, and *ω* is the weight coefficient:

$$\begin{array}{c} \begin{array}{cc} E^{GIF} & =\lambda_{1}\int_{\Omega }{\left|I_{0}-c_{1}\right|}^{2}H_{\varepsilon }(\phi )dx\\ & +\lambda_{1}\int_{\Omega }{\left|I_{0}-c_{1}\right|}^{2}(1-H_{\varepsilon }(\phi ))dx \end{array} \end{array}$$ (12)

LGIF improves the segmentation accuracy of LBF and adds robustness, to introduce a new way to segment images. However, its weights are set manually, which makes it weaker for applications.

## 3 Proposed method

Enlightened by the previous work above, a hybrid model based on level sets is proposed, with the energy function defined as:

$$\begin{array}{c} \begin{array}{c} E=\omega E^{G}+(1-\omega )E^{L}+E^{R} \end{array} \end{array}$$ (13)

In (13), *E*^*G*^ is the global and *E*^*L*^ the local fitting components, and *E*^*R*^ is the regularization term. *ω* ∈ [0, 1] controls the significance of the global vs. local components during contour evolution. *ω* can be tuned according to the degree of gray scale inhomogeneity. The more homogenous the image is, the greater the value of *ω* is; i.e., the more dominant the global driving is. On the contrary, the higher the degree of gray scale imbalance, the less the value of omega is; i.e., the more dominant the local driving is.

Herein, the simplified form of fitting in the C-V model serves as *E*^*G*^. [32] pointed out that the numerical calculation of (5) is unstable, which led to complex implementation. Thus, we derive a simplified form of (5). As shown in [20], the main forces driving the evolution of the level set are −λ_1_(*I*_0_ − *c*_1_)^2^ + λ_2_(*I*_0_ − *c*_2_)^2^. Therefore, we set λ_1_ = λ_2_ = 1, and convert it to $2\left(c_{1}-c_{2}\right)\left(I_{0}-\frac{c_{1}+c_{2}}{2}\right)$ by using the squared difference. Furthermore [37], pointed out that in the process of level set evolution, the hard threshold $\frac{c_{1}+c_{2}}{2}$ determines each pixel on the change of the level set function *ϕ*. Thus, 2(*c*_1_ − *c*_2_) can be set to a constant. To facilitate the global fitting term, we set 2(*c*_1_ − *c*_2_) = 1, then put it into the energy function to obtain the reduced global fitting as:

$$\begin{array}{c} \begin{array}{c} E^{G}(c_{1},c_{2},\phi )=\int_{\Omega }(I_{0}-\frac{c_{1}+c_{2}}{2})H_{\varepsilon }(\phi (x))dx \end{array} \end{array}$$ (14)

**Fig 1:**
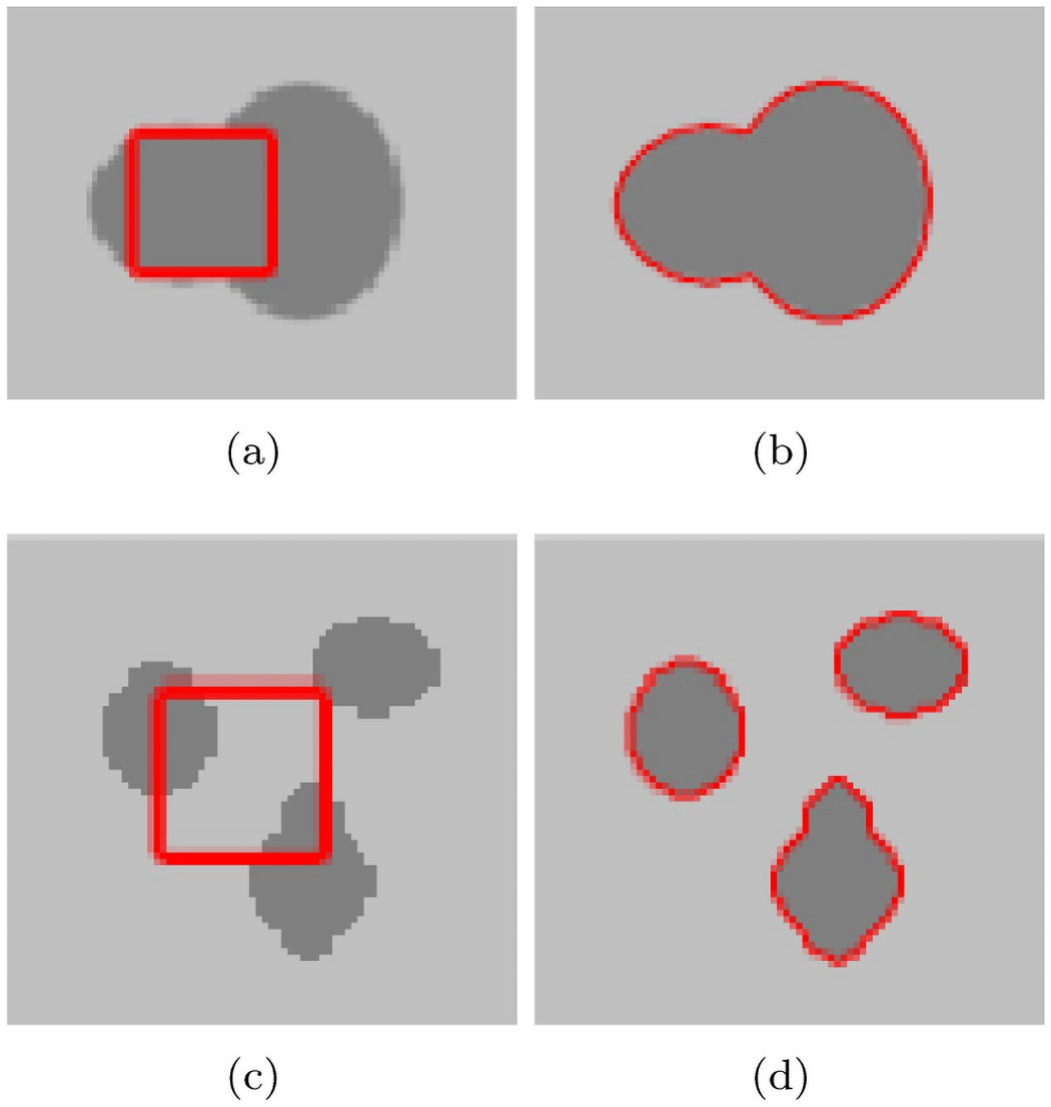
The segmentation results of simple image using (14).

**Fig 2:**
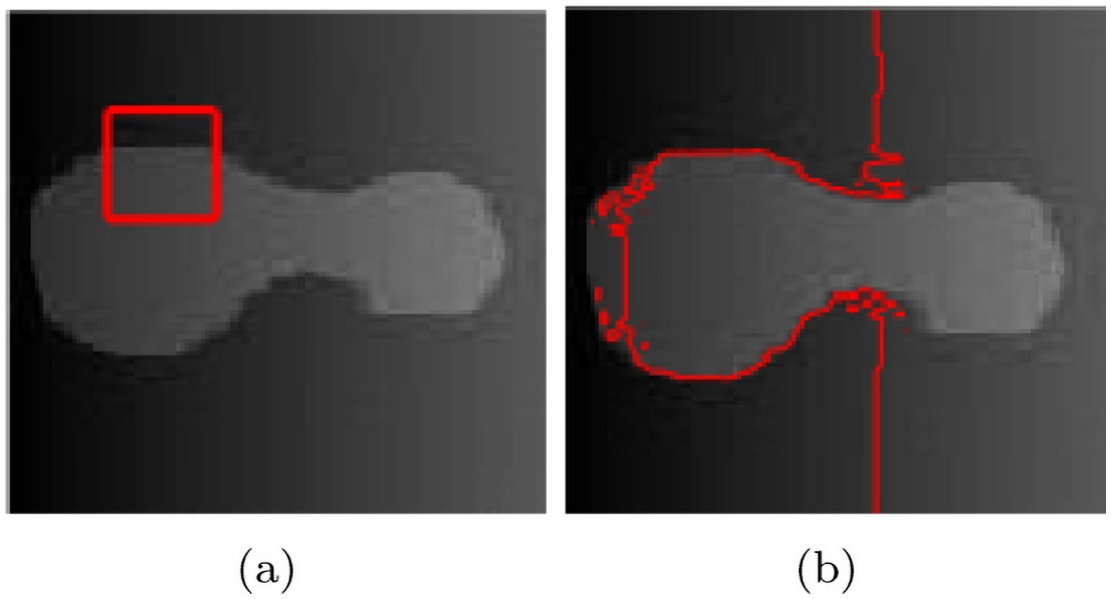
The segmentation result of uneven intensity image using (14).

When solving (14), a Hamilton-Jacobi differential equation can be obtained, whose speed is $I_{0}-\frac{c_{1}+c_{2}}{2}$. According to the evolution law of Hamilton-Jacobi differential equation, when the velocity is greater than zero, the contour moves along the direction opposite to the normal; otherwise, it moves along the normal direction. The driving force of the contour is simple, which can accelerate the evolution of the contour, and has a good segmentation effect for simple homogeneous images. Fig 1 shows the results of the simplified model on the segmentation of the synthesized simple image. However, when the image is not uniform, the target cannot be obtained correctly, as shown in Fig 2.

Next, we define the local fitting term:

$$\begin{array}{c} \begin{array}{cc} \; & E^{L}(k_{s}(x),k_{l}(x),\phi )\\ & =\int \int G_{\sigma }*{\left|I_{0}-k_{s}(x)\right|}^{2}H_{\varepsilon }(\phi (y))dydx\\ & +\int \int G_{\sigma }*{\left|I_{0}-k_{l}(x)\right|}^{2}(1-H_{\varepsilon }(\phi (y)))dydx \end{array} \end{array}$$ (15)

where *G*_*σ*_ refers to the weighted Gaussian function on gray scale and distance, namely weighted bilateral filtering [38], shown as follows:

$$\begin{array}{c} \begin{array}{c} \left\{ \begin{array}{c} G_{\sigma }=\frac{1}{{\left(2\pi \right)}^{1/2}\sigma }e_{1}e_{2}\\ e_{1}=e^{-\frac{{\left(x_{i}-x_{c}\right)}^{2}+{\left(y_{i}-y_{c}\right)}^{2}}{2\sigma^{2}}}\\ e_{2}=e^{-\frac{{\left(gray(x_{i},y_{i})-gray(x_{c},y_{c})\right)}^{2}}{2\sigma^{2}}} \end{array}\right. \end{array} \end{array}$$ (16)

**Fig 3:**
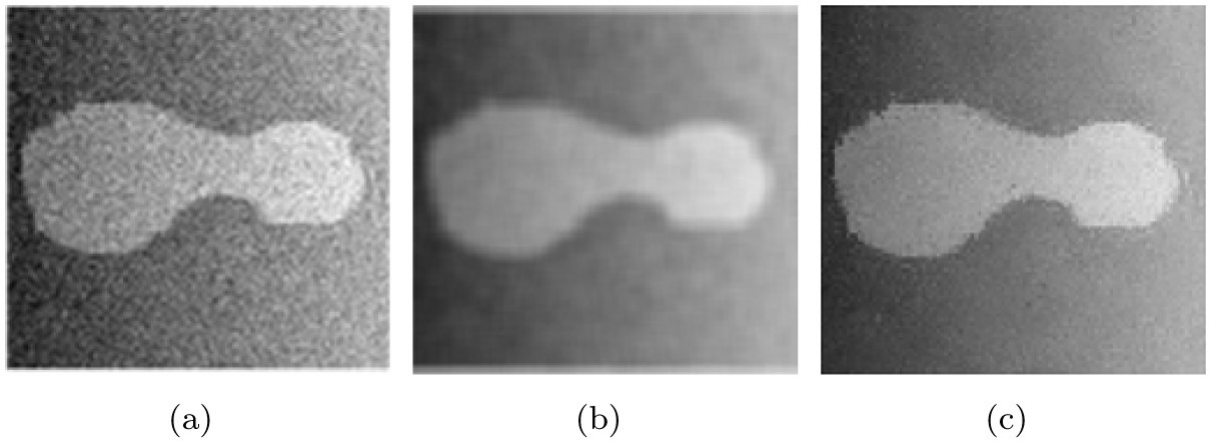
Comparison between the Bilateral filtering and the Gaussian filtering for a synthetic image. (a) Original image. (b) The Gaussian filtering. (c) The Bilateral filtering.

This function takes into account both spatial distance and image value differences, so that a point far away from the edge will only slightly affect pixel values on edges. As a result, it helps suppress noise and retain boundaries as well. Take the composite image in Fig 3 for example, Fig 3(a) contains a lot of noise and the gray level is not uniform. Fig 3(b) and 3(c) show images optimized by using the same template with unweighted Gaussian filtering and weighted Gaussian filtering proposed in (16) respectively. It can be seen, in Fig 3(b), the target object becomes blurred, while in Fig 3(c) not only the noise is removed, but the target area is enhanced.

Ω_*k*_ of window size *k* is the neighborhood of (*x*_*i*_, *y*_*i*_), we have

$$\begin{array}{c} \begin{array}{c} \left\{ \begin{array}{c} k_{m}(x)=mean(I(x)|x\in \Omega_{k})\\ k_{s}(x)=mean(I(x)|x\in \Omega_{s})\\ k_{l}(x)=mean(I(x)|x\in \Omega_{l}) \end{array}\right. \end{array} \end{array}$$ (17)

where

$$\begin{array}{c} \begin{array}{c} \left\{ \begin{array}{c} \Omega_{s}=\{y|I(y)<k_{m}(x)\}\cap \Omega_{k}\\ \Omega_{l}=\{y|I(y)>k_{m}(x)\}\cap \Omega_{k} \end{array}\right. \end{array} \end{array}$$ (18)

**Fig 4:**
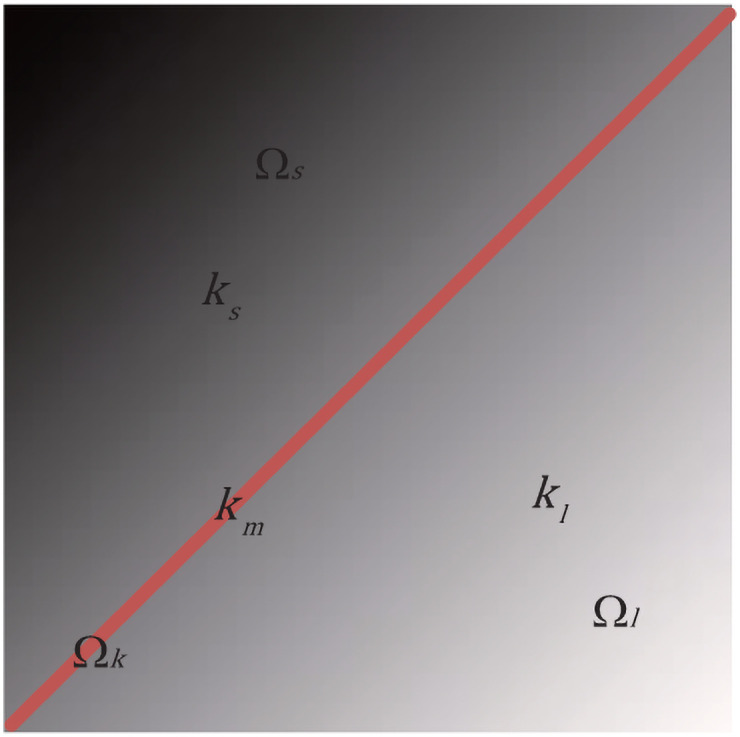
Illustration of regions Ω_*k*_, Ω_*s*_ and Ω_*l*_.

According to Eqs (17) and (18), for a given square Ω_*k*_, once *k*_*m*_ is calculated directly, the area Ω is split into two parts; i.e. Ω_*s*_ and Ω_*l*_, in the light of the gray value in relation to *k*_*m*_. This is shown in Fig 4.

In addition, in order to keep the equation stable during evolution and avoid reinitialization, a regularization term is added:

$$\begin{array}{c} \begin{array}{c} E^{R}(\phi )=u\int_{\Omega }\delta_{\varepsilon }(\phi )|\nabla \phi |dx+v\int_{\Omega }\frac{1}{2}{\left(|\nabla \phi |-1\right)}^{2}dx \end{array} \end{array}$$ (19)

To sum up, for a given image *I*_0_ ∈ *R*^2^(Ω), the ultimate energy function of level set is formulated as:

$$\begin{array}{c} \begin{array}{cc} \; & E(c_{1},c_{2},k_{s},k_{l},\phi )=\omega \int_{\Omega }(I_{0}-\frac{c_{1}+c_{2}}{2})H_{\varepsilon }(\phi )dx\\ & +(1-\omega )\int \int G_{\sigma }*{\left|I_{0}-k_{s}(x)\right|}^{2}H_{\varepsilon }(\phi (y))dydx\\ & +\left(1-\omega \right)\int \int G_{\sigma }*{\left|I_{0}-k_{l}(x)\right|}^{2}(1-H_{\varepsilon }(\phi (y)))dydx\\ & +u\int_{\Omega }\delta_{\varepsilon }(\phi )|\nabla \phi |dx+v\int_{\Omega }\frac{1}{2}(|\nabla \phi |-1{)}^{2}dx \end{array} \end{array}$$ (20)

where *ω* ∈ [0, 1]. When *ω* = 1, the model has no local fitting term and degenerates into the simplified form of C-V model with a regularization term. Minimizing the function *E*, the evolution equation can be computed as:

$$\begin{array}{c} \begin{array}{cc} \frac{\partial \phi }{\partial t} & =-\delta_{\varepsilon }(\phi )[\omega e_{c}+(1-\omega )(e_{s}(x)-e_{l}(x))]\\ & +u\delta_{\varepsilon }(\phi )\text{div}(\frac{\nabla \phi }{\left|\nabla \phi \right|})+v(\nabla^{2}\phi -\text{div}(\frac{\nabla \phi }{\left|\nabla \phi \right|})) \end{array} \end{array}$$ (21)

where *δ*_*ε*_ is defined in (3), *e*_*c*_, *e*_*s*_ and *e*_*l*_ are presented as:

$$\begin{array}{c} \begin{array}{c} \left\{ \begin{array}{c} e_{c}=I_{0}-\frac{c_{1}+c_{2}}{2}\\ e_{s}(x)=\int_{\Omega }G_{\sigma }(x-y)|I_{0}-k_{s}(x){|}^{2}dydx\\ e_{l}(x)=\int_{\Omega }G_{\sigma }(x-y)|I_{0}-k_{l}(x){|}^{2}dydx \end{array}\right. \end{array} \end{array}$$ (22)

Applying the finite difference method to discretize: (21):

$$\begin{array}{c} \begin{array}{c} \phi_{i,j}^{n+1}=\phi_{i,j}^{n}+\Delta t(\omega A_{i,j}+(1-\omega )B_{i,j}+C_{i,j}) \end{array} \end{array}$$ (23)

where *A*_*i*,*j*_, *B*_*i*,*j*_ and *C*_*i*,*j*_ are calculated as:

$$\begin{array}{c} \begin{array}{cc} \; & A_{i,j}=-\delta_{\varepsilon }(\phi_{i,j})(I_{0}(i,j)-\frac{c_{1}(\phi_{i,j}^{n})+c_{2}(\phi_{i,j}^{n})}{2})\\ & B_{i,j}=-\delta_{\varepsilon }(\phi_{i,j})(e_{s}(x)-e_{l}(x))\\ & C_{i,j}=u\delta_{\varepsilon }(\phi_{i,j})\text{div}(\frac{\nabla \phi_{i,j}}{\left|\nabla \phi_{i,j}\right|})+v(\nabla^{2}\phi_{i,j}-\text{div}(\frac{\nabla \phi_{i,j}}{\left|\nabla \phi_{i,j}\right|})) \end{array} \end{array}$$ (24)

where Δ*t* is the iteration step.

The segmentation procedure of the proposed method can be summarized as:

Step 1: Set parameters, to define the initial contour C, initialize *ϕ*_0_.
Step 2: Calculate *c*_1_ and *c*_2_ via (4), and calculate *k*_*s*_ and *k*_*l*_ via (17).
Step 3: Update the level set function via (21).
Step 4: Judge whether convergence is achieved. If the stability condition is reached, stop iteration and obtain the segmentation result. If not, go to Step 2.

## 4 Experimental results and analysis

**Fig 5:**
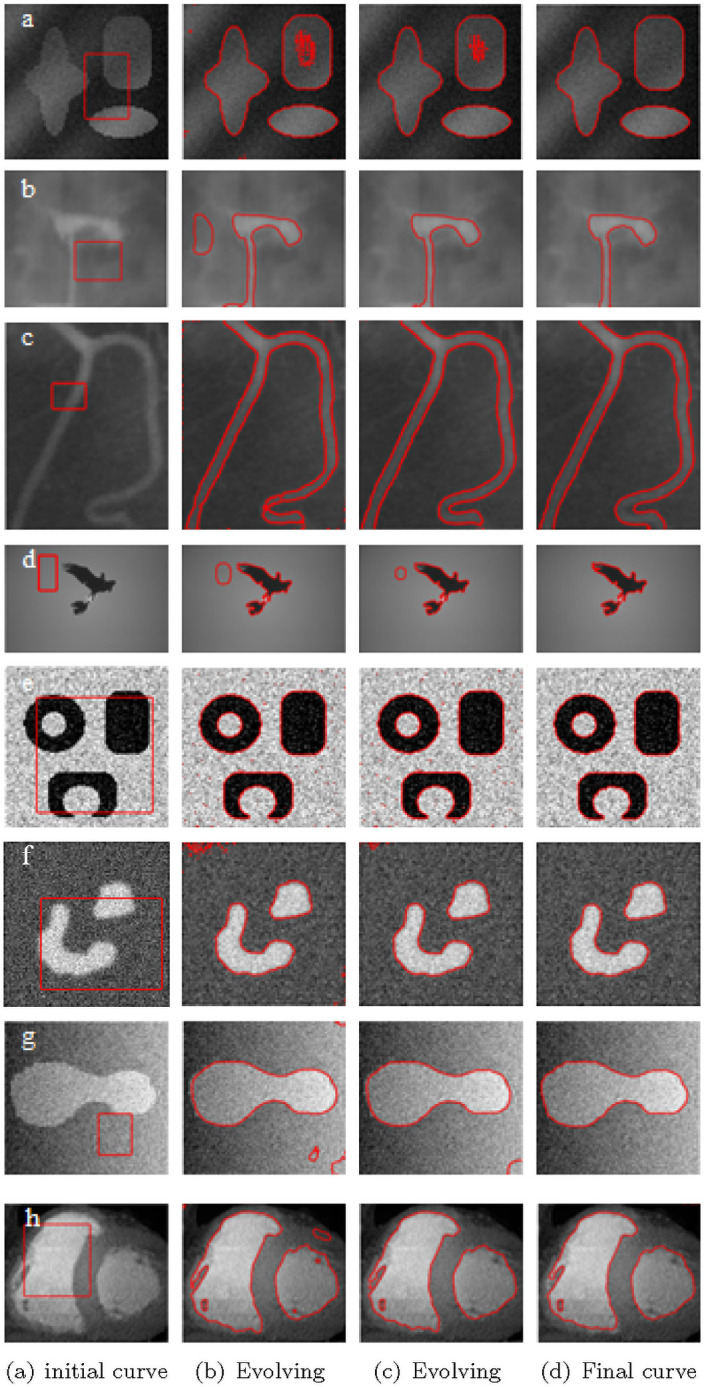
Segmentation results of partial images. column 1: initial contour. column 2 to 3: intermediate segmentation results. column 4: final segmentation results.

In order to verify the effectiveness of the proposed algorithm, this section presents experimental results and comparisons to related methods. The experimental environment include: CPU i5 Gen, 8GB running memory, Windows 10 64-bit operating system, MATLAB R2016a. In the experiments, the initial level set function *ϕ*_0_ is set to a small constant function *ϕ*_0_ = *c*_0_. If there is no special explanation, the parameters are set as: *c*_0_ = 2, *μ* = 0.01 × 255^2^, *v* = 2, *ε* = 2, Δ*t* = 0.1, *k* = 13 and *σ* = 2. The weight coefficient *ω* is adjusted according to the complexity of the image. For images with high noise and contrast, *ω* is greater than 0.5 to ensure the evolution rate of the image. For low-contrast images, the *ω* is less than 0.5. Fig 5 shows the segmentation process and results for different images.

In Fig 5, the first row shows composite images, with heavy shadows. The second row shows salpingography images, with low contrast. The third row shows real images of blood vessels, with inhomogeneous intensities. The fourth row shows images of birds with uneven gray scales. The fifth and sixth rows are composite images with noises. The seventh row displays the composite noisy image with uneven illumination. The images in the last row are of a real heart. These images are characterized by low contrast, high noise and blurred edges. It is clear that the algorithm proposed extract exact boundaries, which are in line with visual judgements. Some parameters are listed in Table 1. Others are default values mentioned above.

**Table 1:** The permissible range of length term *μ*, *k* and *ω* used in Fig 5.

image	*μ*	*k* * *k*	*ω*
a	0.01*255*255	9*9	0.2
b	0.005*255*255	13*13	0.2
c	0.005*255*255	21*21	0.2
d	0.02*255*255	9*9	0.1
e	0.01*255*255	13*13	0.1
f	0.01*255*255	13*13	0.7
g	0.3*255*255	21*21	0.3
h	0.05*255*255	17*17	0.2

**Fig 6:**
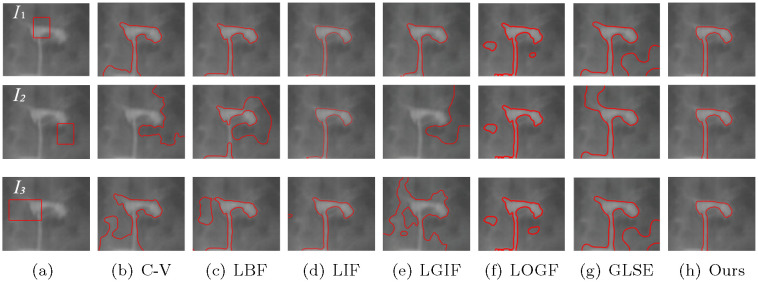
Comparisons for a tubal angiography image with varying initial contours.

**Fig 7:**
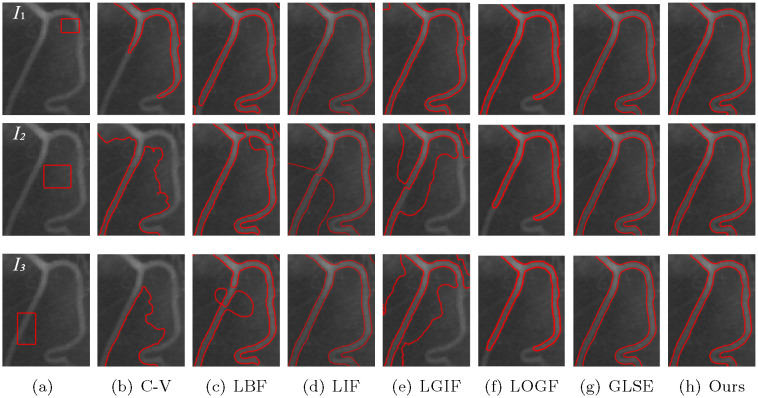
Comparisons for a vessel image with variying initial contours.

**Fig 8:**
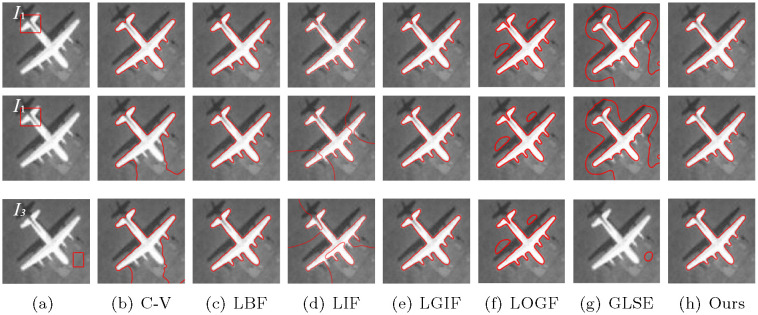
Comparisons for an airplane image with varying initial contours.

**Fig 9:**
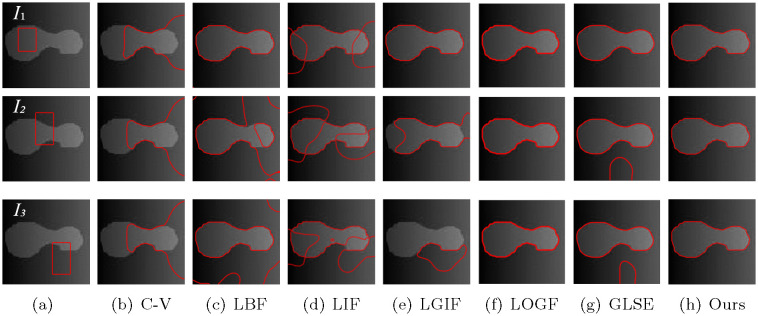
Comparisons for a synthetic image with varying initial contours.

In addition, in order to verify the insensitivity of the model to the initial contour, we set different initial contour positions on a number of images without changing other parameters, and compare the models mentioned above, including C-V, LBF, LIF, LGIF, LOGF and LGSE model. In the C-V model, λ_1_ = λ_2_ = 1. The C-V model does not require any more parameters, which is one of its advantages. In the LBF model, λ_1_ = λ_2_ = 1, *v* = 1 and *ε* = 1. In LIF model, *c*_0_ = 2 and *ε* = 1. In the LGIF model, *v* = 1, *ε* = 1, λ_1_ = λ_2_ = 1. In LOGF model, *ω* = 10, *v* = 1, *ε* = 1, λ_1_ = λ_2_ = 1. In GLSE model, *v* = 1, *ε* = 1.5 and λ_1_ = λ_2_ = 1. Figs 6–9 show the segmentation results of low contrast images for each module with different initial contours.

In Fig 6, under the premise of initial contour outlined in first row, the results of LBF, LIF, LGIF and the proposed method are close, while the results of C-V and GLSE are not ideal. In the case of three different initial contours, The three results are not very different in the LIF, LOGF model and the proposed method, that indicate these three models are insensitive to the position of the initial contour. In contrast, C-V, LBF, LGIF and GLSE obtained different results under the three initial contours, indicating that the results are easily affected by the initial contour.

Fig 7 is a real blood vessel image with low contrast. Similarly, after adjusting the parameters with the initial contour *I*_1_, each model achieved its best segmentation results. When other parameters are unchanged, only the initial contour is changed, and the segmentation results are changed accordingly. However, GLSE and our model does not have such a problem.

Fig 8 experiments on a complex background with shadows. The LBF and LGIF models and the model presented in this paper get the target under three different contours. The C-V and LIF models split the target only under contour *I*_1_, However, no matter how LOGF and GLSE model adjust parameters, the target is not segmented.

Fig 9 is a composite image with inhomogeneous gray scales. Both the LOGF model and the model presented in this paper obtain satisfactory results under three initial contours, while the LBF, LGIF, GLSE model only gets correct results under the initial contour *I*_1_.

Through experimental results comparison with other models in Figs 7–9, the proposed algorithm prevails in different scenarios under the premise of any initial contours. in other models, the change of the initial contour will lead to the error of segmentation results. some of the parameters, such as scale parameter and length term coefficient, must be adjusted in order to get the correct result, and this is a complex process.

In Table 2, under the condition of the first initial profile, iterations and execution times are listed. Note that we only list the time and number of iterations in the case of the initial contour *I*_1_, because from the visual point of view, the segmentation result of the first initial contour is the best. The GLSE model has the longest segmentation time, because it needs to calculate global variance and local variance to balance the weight of global and local items in each iteration. The C-V model has the largest number of iterations due to the use of global information. The segmentation time of LIF model is relatively short because of the low computation cost in each iteration. In the LGIF model, the segmentation time is affected by the weight between the local energy and the global energy. For Fig 9, the time taken is very short due to the appropriate weight *ω*. However, for Figs 6, 7 and 8, the segmentation time is relatively long under this weight *ω*. The LBF model needs more segmentation time and fewer number of iterations than the LIF model and the LGDF model for these images. The LOGF model is affected by the optimized LoG energy term, and the segmentation time and iteration number are higher than that of LBF model in Fig 6, and lower than that of LBF model in Fig 9. The proposed method has close to the least number of iterations and least execution time with the exception of Fig 6. Note that only the proposed algorithm successfully extracts fine boundaries.

**Table 2:** Iterations \ execution time(s) of Figs 6–9.

Model	Fig 6 iterations\time(s)	Fig 7 iterations\time(s)	Fig 8 iterations\time(s)	Fig 9 iterations\time(s)
C-V	500\0.9420	500\1.8316	1700\2.6685	400\1.1526
LBF	20\0.2527	60\0.8329	8\0.9871	24\0.4190
LIF	120\0.1382	30\0.5277	30\0.6353	10\0.1949
LGIF	220\0.8472	70\0.5158	60\0.6606	40\0.2778
LOGF	150\0.6355	100\0.4745	100\0.5327	10\0.3511
GLSE	130\2.3409	150\2.2141	200\2.1203	20\0.5237
Ours	400\0.7041	20\0.1403	6\0.0696	24\0.0918

Naturally, Jaccard similarity coefficient (*JSC*) and Dice similarity coefficient (*DSC*) [39] are popular to quantitatively evaluate the performance of segmentation results. These are defined below:

$$\begin{array}{c} \begin{array}{cc} JSC & =\frac{N(S_{g}\cap S_{m})}{N(S_{g}\cup S_{m})}\\ DSC & =\frac{2N(S_{g}\cap S_{m})}{N(S_{g})\cup N(S_{m})} \end{array} \end{array}$$ (25)

where *S*_*g*_ represents the ground truth, and *S*_*m*_ represents the segmented regions. When the *JSC* and *DSC* are closer to 1, the image segmentation is better. Specifically *JSC* = *DSC* = 1, means that the detected region is identical to the ground truth.

**Fig 10:**
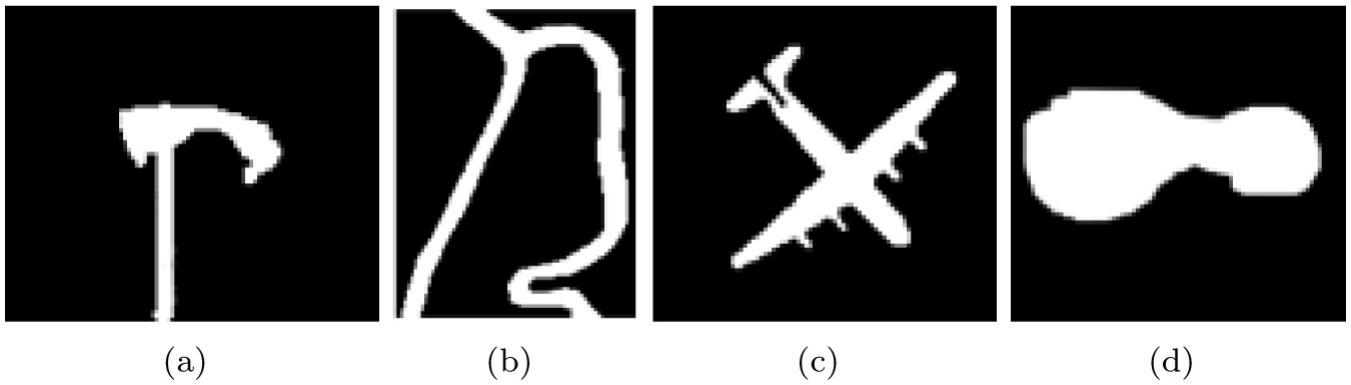
Ground truth invented mannually of (a) Fig 6, (b) Fig 7, (c) Fig 8 and (d) Fig 9.

Table 3 demonstrates the *JSC* and *DSC* of Figs 6–9. The *JSC* and *DSC* is in the case of the initial contour *I*_1_, The ground truth are obtained by manual segmentation, see Fig 10.

**Table 3:** The *JSC* and *DSC* of different models for Figs 6–9.

Image	Fig 6 *JSC*\*DSC*	Fig 7 *JSC*\*DSC*	Fig 8 *JSC*\*DSC*	Fig 9 *JSC*\*DSC*
C-V	0.6124\0.6523	0.6363\0.7810	0.9793\0.9854	0.6566\0.7541
LBF	0.7256\0.8410	0.9187\0.9518	0.9849\0.9920	0.9864\0.9931
LIF	0.8134\0.8912	0.9513\0.9750	0.9816\0.9913	0.6910\0.8173
LGIF	0.8136\0.8970	0.8810\0.9320	0.9822\0.9925	0.9524\0.9701
LOGF	0.7709\0.8706	0.8904\0.9420	0.7162\0.8346	0.9825\0.9912
GLSE	0.5252\0.6887	0.9560\0.9775	0.5112\0.6765	0.9833\0.9915
Ours	0.8692\0.9319	0.9655\0.9824	0.9831\0.9915	0.9872\0.9931

**Fig 11:**
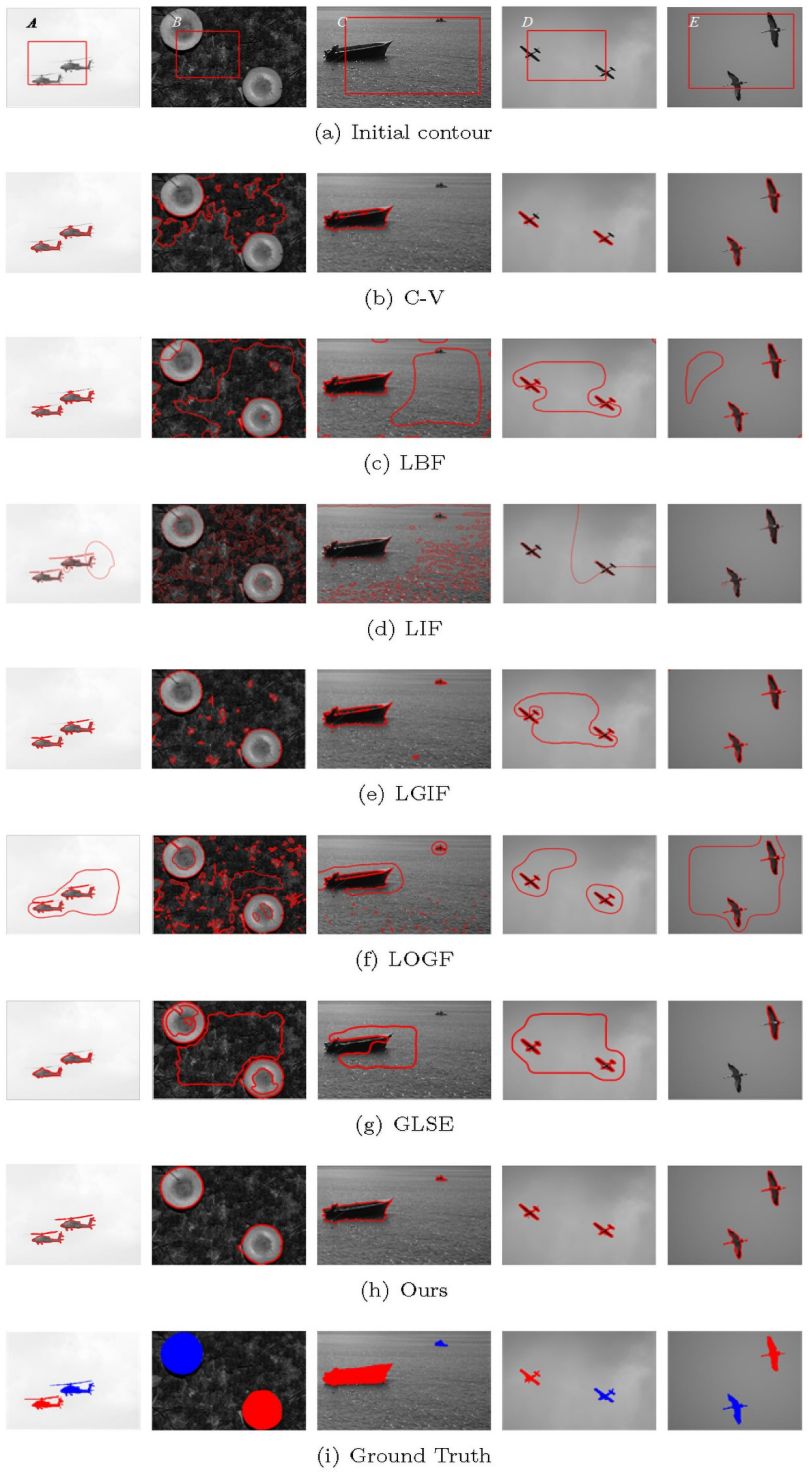
Experiments on standard database.

**Fig 12:**
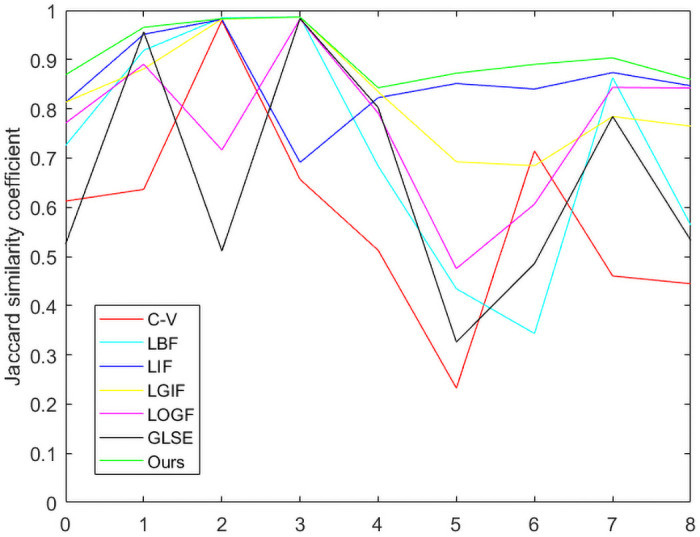
Comparison of various models in terms the *JSC*.

**Fig 13:**
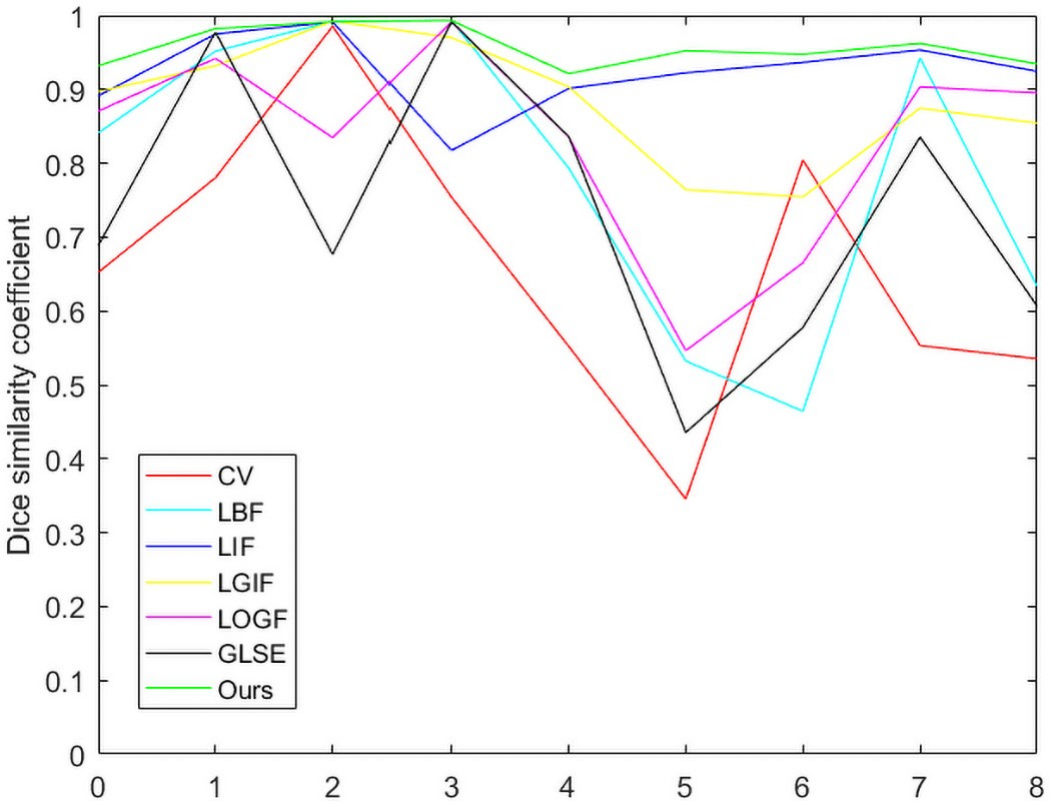
Comparison of various models in terms the *DSC*.

Table 4 shows the *JSC* and *DSC* coefficients of Fig 11, whose images are taken from the Weizmann database [40]. In addition, *JSC* and *DSC* in Tables 3 and 4 are represented by broken line graphs, As shown in Figs 12 and 13.

**Table 4:** The *JSC* and *DSC* of different models for Fig 11.

Image	A JSC\DSC	B JSC\DSC	C JSC\DSC	D JSC\DSC	E JSC\DSC
C-V	0.5124\0.5523	0.2324\0.3454	0.7142\0.8042	0.4603\0.5532	0.4445\0.5354
LBF	0.6834\0.7934	0.4343\0.5322	0.3432\0.4642	0.8632\0.9426	0.5632\0.6332
LIF	0.8226\0.9013	0.8513\0.9224	0.8402\0.9365	0.8736\0.9534	0.8468\0.9245
LGIF	0.8345\0.9034	0.6922\0.7643	0.6844\0.7545	0.7843\0.8745	0.7646\0.8544
LOGF	0.7911\0.8356	0.4753\0.5464	0.6062\0.6654	0.8435\0.9034	0.8422\0.8952
GLSE	0.8024\0.8364	0.3262\0.4354	0.4854\0.5778	0.7843\0.8355	0.5325\0.6065
Ours	0.8426\0.9213	0.8723\0.9524	0.8902\0.9475	0.9035\0.9623	0.8595\0.9345

As can be seen from Figs 12 and 13, the C-V model with global information has the worst segmentation results for Figs 6–11. However, the segmentation results of LIF model, LGIF model and the proposed model is relatively stable. The segmentation results of LBF model, LOGF model and GLSE model fluctuate greatly.

To sum up, in comparison with the other six models, our method can better balance segmentation accuracy and efficiency. It requires less time and iterations; it is not sensitive to the initial contour; and it improves the C-V, LBF and LGIF models and substantially enhances their accuracy and efficiency.

## 5 Conclusion

For accurate segmentation in inhomogeneous images and fast evolution iterations, we proposed an improved active contour model. According to the curve evolution theory, the C-V model is simplified. At the same time, new local and global fitting term are incorporated to build a new energy function, which helps in image segmentation for sophisticated applications. Furthermore, our method is simple to initialize, takes less time to calculate, converges faster iteratively, and is more robust to pixel perturbations. Experiments and subjective assessment indices proved the effectiveness and efficiency of our approach.
